# *De curandis hominum morbis*: una receta médica del siglo XVIII para el sarampión y las viruelas en el Nuevo Reino de Granada

**DOI:** 10.7705/biomedica.4995

**Published:** 2020-06-30

**Authors:** Alejandra Lozano, Julio César Martínez, Jorge Uribe, Alberto Gómez, Sneider Alberto Figueredo, Ignacio Briceño

**Affiliations:** 1 Grupo de Genética Humana, Universidad de La Sabana, Chía, Colombia Universidad de la Sabana Grupo de Genética Humana Universidad de La Sabana Chía Colombia; 2 Facultad de Ciencias Sociales, Pontificia Universidad Javeriana, Bogotá, D.C, Colombia Pontificia Universidad Javeriana Pontificia Universidad Javeriana BogotáD.C, Colombia; 3 Instituto de Genética Humana, Facultad de Medicina, Pontificia Universidad Javeriana, Bogotá, D.C., Colombia Pontificia Universidad Javeriana Instituto de Genética Humana Pontificia Universidad Javeriana BogotáD.C Colombia

**Keywords:** historia de la medicina, sarampión, viruela, registros médicos, historia natural, prescripciones, medicina tradicional, History of medicine, measles, smallpox, medical records, natural history, prescriptions, medicine, traditional

## Abstract

En el Archivo Histórico de la Biblioteca “Octavio Arizmendi Posada” de la Universidad de La Sabana, se encuentra una colección de más de un centenar de recetas médicas de finales del siglo XVIII donadas por el presbítero Cipriano Rodríguez Santa María, epónimo institucional del archivo. Estos textos son un legado histórico médico y un fundamento para comprender la terapéutica colonial y tradicional de diversas enfermedades. En este artículo, se describen algunas recetas para el tratamiento de la viruela y el sarampión, como aporte a la historia de la medicina en Colombia.

## Abstract (en)

A collection of more than one hundred medical recipes from the late 18th century was donated by Presbyter Cipriano Rodríguez Santa María, institutional eponym of the collection at the Historical Archive “Octavio Arizmendi Posada” of the library of the *Universidad de La Sabana* in Colombia. These texts represent an important historical and medical legacy and they constitute an important basis for understanding the colonial and traditional therapeutics related to various diseases. In this article, we describe one of these recipes for the treatment of smallpox and measles as a contribution to the history of medicine in Colombia.

Hasta el siglo XVII, la medicina continuaba siendo una ciencia poco desarrollada a pesar de los grandes avances alcanzados por la humanidad en varios campos del conocimiento. No obstante, a partir de la segunda mitad de ese siglo y hasta comienzos del XIX, surgieron varias teorías médicas que entrarían a disputar un lugar preeminente frente a las ideas de Galeno (130-210 d. C.), cuya vigencia sobrepasaba los 1.500 años. Dichas teorías se enmarcaron en sistemas médicos conocidos como la yatroquímica, la yatromecánica, el animismo y el vitalismo, el solidismo, el brownismo, el mesmerismo, etc., escuelas de pensamiento terapéutico que dieron lugar a diferentes conceptos de enfermedad y, por consiguiente, a la modificación de los tratamientos utilizados hasta entonces. Cabe destacar la gran influencia que, en esos años, tuvieron las diferentes corrientes del pensamiento filosófico en la evolución de la ciencia médica de la época. Asimismo, deben considerarse hechos de importancia histórica como la Revolución Francesa, que sirvieron de contexto y tuvieron impacto en la sociedad del siglo XVIII, preparando, estimulando y, finalmente, consiguiendo la transformación científica de la medicina.

La medicina antigua recurría al uso de productos de la naturaleza, y los tratamientos se basaban en conocimientos botánicos y naturistas con poco fundamento médico, especialmente sobre las bases fisiopatológicas y farmacológicas de las enfermedades. En el caso del Virreinato de la Nueva Granada, los documentos históricos dan fe de ello al reseñar el uso de recetas médicas y tratamientos elementales elaborados con productos naturales: plantas, agua y algunos minerales.

El conocimiento sobre las propiedades de los productos naturales se vio impulsado por la Expedición Botánica del Nuevo Reino de Granada, cuyo fin fue el estudio de los recursos naturales para su aprovechamiento en la ciencia. El director de la expedición fue José Celestino Mutis (1732-1808), quien viajó al Virreinato de la Nueva Granada como médico del Virrey Pedro Messía de la Cerda (1700-1783). Tras terminar el mandato del virrey en 1772, Mutis decidió permanecer en el territorio que lo había recibido en 1760, donde promovería nuevos tratamientos con base en los avances de la ciencia europea, incluida, por ejemplo, la variolización ^1^^,^^2^.

La viruela ha sido una de las enfermedades más devastadoras en la historia de la humanidad. Su erradicación en 1979, después de un exitoso programa de vacunación mundial, fue una de las victorias más importantes de la medicina moderna. Esta enfermedad infecciosa tuvo un terrible impacto en las poblaciones, a tal punto que suele mencionársela en muchos de los eventos históricos más destacados de la historia antigua. La infección viral recibe su nombre del término en latín *variola*, que hace referencia a las pústulas que aparecen en el cuerpo de los infectados. Era una enfermedad con una tasa muy elevada de morbimortalidad, con graves secuelas, entre ellas, ceguera, esterilidad y cicatrices en la piel. Muy contagiosa, se transfería por contacto directo con los fluidos corporales de los enfermos o, indirectamente, por el contacto con objetos contaminados ^3^.

La infección es causada por un virus de la familia Poxviridae, el cual es relativamente grande y de forma rectangular, con una membrana externa compuesta por lipoproteínas. Está codificado por un ADN de doble cadena resguardado en una gruesa capa, lo que le confiere gran resistencia y poder patógeno ^4^. El virus ingresa al organismo por las membranas mucosas del sistema respiratorio superior donde prolifera y, después, se desplaza por el sistema linfático hasta ingresar al torrente sanguíneo. Eventualmente, el virus invade la epidermis, lo que causa el síntoma más visible de la enfermedad: las pústulas. Los pacientes con viruela se caracterizaban por presentar sarpullido y lesiones en la piel que se hacían visibles simultáneamente con las lesiones en la orofaringe ^5^.

El flagelo del sarampión ha sido algo más benigno que la viruela, pues su mortalidad es menor. El virus que lo causa es esférico y su genoma contiene ARN de cadena sencilla, pertenece al género *Morbillivirus* de la familia Paramyxoviridae y su transmisión provoca una infección muy contagiosa que no tuvo contención hasta que se inició la aplicación de la vacuna elaborada a partir de virus vivos atenuados ^6^.

Hasta la fecha, el tratamiento de estas virosis no comprende estrategias diferentes a la administración de antipiréticos para controlar la fiebre y, en el caso del sarampión, de antitusígenos para moderar la tos. Antes de la introducción de las vacunas, el único recurso eran los tratamientos naturistas, como el que se presenta a continuación.

En la figura 1, se observa uno de los folios manuscritos en los que se detalla la receta para el sarampión y las viruelas, el cual se conserva en el Archivo Histórico “Cipriano Rodríguez Santamaría”, Fondo Manuel María Mosquera, de la Biblioteca “Octavio Arizmendi Posada” de la Universidad de La Sabana (Chía, Colombia). El documento llegó a manos del presbítero Rodríguez Santa María por estar emparentado con la familia Mosquera de Popayán, aunque no se especifica quién fue su propietario en dicha ciudad, ni el facultativo que la redactó. Tanto esta como las demás recetas conservadas en el archivo se pueden consultar en línea en https://intellectum.unisabana. edu.co/handle/10818/18140, parte 4.

**Figura 1:**
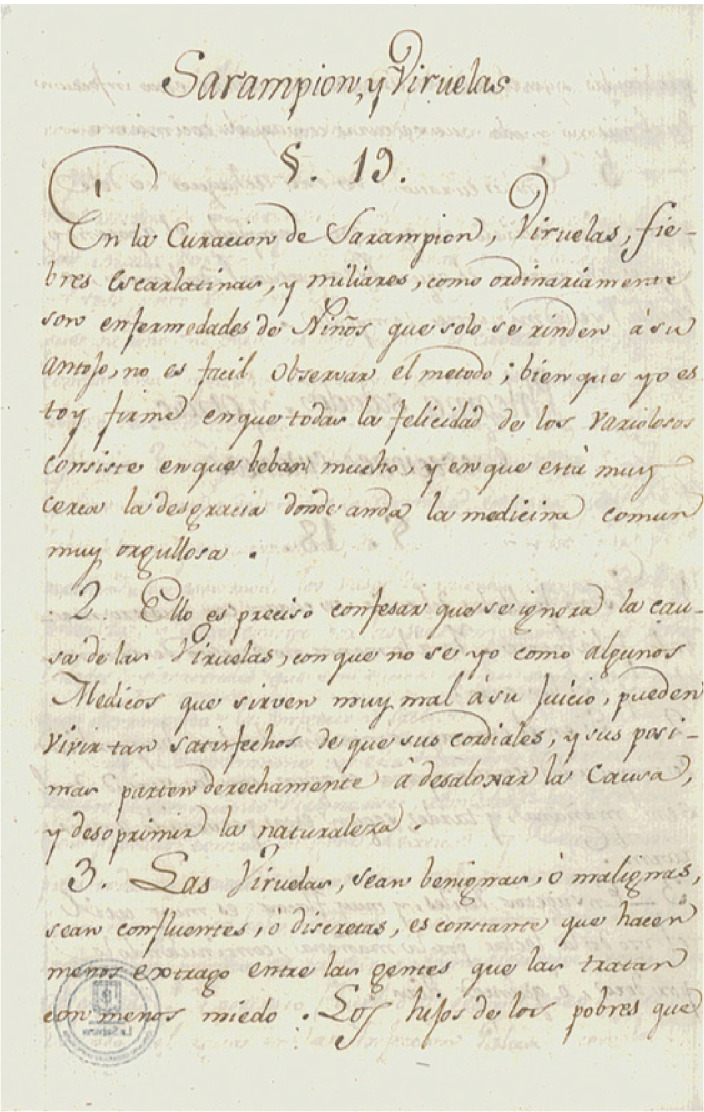
Receta para el sarampión y las viruelas

Fuente: Universidad de La Sabana, Archivo Histórico Cipriano Rodríguez Santa María, Fondo Manuel María Mosquera, caja: 10, carpeta: 2, 111 verso - 113 recto y 116 recto - 120 verso. Archivo completo disponible en: https://intellectum.unisabana.edu.co/handle/10818/18140.Parte 4.pdf

## Transcripción de las recetas

“Sarampión y viruelas - S19

1. En la curación de Sarampión, Viruelas, Fiebres escarlatinas, y miliares, como ordinariamente son enfermedades de niños que solo se rinden a su antojo, no es facil observar el método; bien que yo estoy firme en que toda la felicidad de los variolosos consiste en que beban mucho, y en que está muy cerca la desgracia, donde anda la medicina comun muy orgullosa.
2. Ello es preciso confesar que se ignora la causa de las Viruelas, con que no se yo como algunos Médicos que sirven muy mal a su juicio, pueden vivir tan satisfechos de que sus cordiales y sus posimas parten derechamente a desalojar la causa, y desoprimir la naturaleza.
3. Las Viruelas, sean benignas, o malignas, sean confluentes, o discretas; es constante que hacen menos estrago entre las gentes que las tratan con menos miedo. Los hijos de los pobres que las esperan a cuerpo descubierto, y aguantan su mal trato, sin implorar mas socorro que el que les avisa su apetito, son sin comparación, mas afortunados que los hijos de los Señores cobardemente escondidos entre tapices, rodeados de asistentes, y muy proveidos de cordiales. Alli, porque se fia toda la curación a la naturaleza: aqui porque se desconfia de la naturaleza y se fia de la curacion a la medicina. Pero que cosa mas comun que curarse con solo agua, o con poco mas que agua, las viruelas? Los muchachos, a la verdad, ni quieren, ni piden, ni toman otra cosa por mas ofrecimiento que les hagan. El año pasado de 53, hubo muchos Variolosos en (Alfotrin), y yo no recete más que ahora, á algunos agua sola (tal vez cocida con azogue) i de limon, i el cocimiento blanco, ó el agua natural, con la quarta parte de leche a lo mas Desto í de lo otro segun el gusto de cada uno, con la prevencion de que siempre que lo pidiessen se les diese a beber; y es constante que de tantos Variolosos como hubo solo murió uno, í otro, debiendo advertirse, que la constitución se explicó desde luego con malignidad. Asi pues,
4. En la curacion del Sarampion, Viruelas y fiebres escarlatinas, y miliares (que en realidad solo se diferencia en el nombre) si la naturaleza procede con movimiento moderado, nada tiene que rezetar el Medico. Es prudencia entonces fiar á la naturaleza todo el negocio, dando a beber segun el metodo. Y aunque los muchachos beban con abundancia, y se resistan a tomar caldo, ó chocolate, no por eso se les retire la bebida, que yo aseguro, que no se moriran por esto. He asistido a muchisimos, que en 5, 6 y 8 dias no han querido tomar mas que agua, ni queria yo tomassen otra cosa, y a feé que esta particularidad no desayudo para su curacion. Assi tengo por grandissimo error las amenazas, y el coco con que obligan a que coman a los niños.
5. Quando el movimiento es impetuoso, tiene muy bien lugar la Sangria en el principio de la curacion de las Viruelas y demás fiebres euruptivas; y luego el agua, segun el metodo: quando es tardo es muy favorable el uso de la aloja templada, de fermentaciones, y baños, con más abrigo que el que se requiere por lo comun, para que suavizada la resistencia del sólido pueda extenderse el movimiento asia el ámbito, y sucede la erupción según el deseo.
6. De este modo curo yo las Viruelas, y me persuado que no son curables, las que no se curan de este modo. Sino considerese bien la condicion de estos enfermos, que es lo que toman cada día y hagase de todo un computo con prudencia. Y verán que sola el agua hace en la curación toda la costa. [...]

Receta para las viruelas

Hase hallado una receta de Lugo para las viruelas q, aunq tarde, asido demucha importancia, y dice que aviendo dolor de cabeza y de espalda con calentura son ciertas las viruelas, y manda sangrar del brazo derecho vena de todo el cuerpo y si aprieta la calentura dos veces, y q tome luego lamedor agrio de sidras, limas, ó limones; haserce tomando cantidad de agua, y echarle otro tanto de agrio de lo dicho, y asucar y le hara jarabe, y del ira tomando por todo el día de quando en quando una cucharada = El agua q ade beber cosida sin nada.

Después de salir las viruelas, no se sangre sino el con necesidad de gran calentura, ó q se este ahogando, ó inflamacion grande en el cuerpo, y ventosas saladas, particularmente con los niños, se ade usar en los hombrillos, y puntas de las nalgas, y tambien secas.

Para la garganta q de ordinario padecen, se hase gargarismo de sevada y llanten cosido, ó bledos con la sebada, y después de cosido, hecharle vinagre de castilla y asucar.

Para las narises de aguamiel de avejas cosida, de miel una parte, y de agua siete = los ojos con agua de azafrán de Castilla =

Al cortar las viruelas se manda tener mucho cuidado q no les de el aire tan presto = y se conocera que esten maduras, quando esten blancas hasta la raíz = suelen tardar, ocho, nuebe, y onse diás conforme son = quando se corten las viruelas, se enjuagan con orines, y sal poca, y luego con un paño mandan q se enjuaguen. El comer sea pollos = masamorras de maíz, con guebos, y sin ellos con poca asucar=

Las aiudas hande ser á noche, y mañana de de cosimiento de malvas salvado y sebada con manteca, sal, y asucar.

Agua para beber, ni se les quite, ni se les de mucha. El abrigo no hade ser, ni tampoco q les de el aire = estas viruelas vienen rebueltas con tabardillo y se conbierten en el por algunos descuidos en los viruelientos =

Curacion para las Viruelas y Alfombrilla q todo el uno aunq mas maliciosa la alfombrilla.

Conocese si son viruelas las q quieren dar, a qualquiera Enfermo, en que abrá calentura con dolor de cabesa y dolor de garganta, y de rabadilla, y á muchos les duelen las espaldas, y les da comezon en las orejas, y en aviendo estas señales, ó qualquiera de ellas son ciertas las viruelas.

Ayudas =

La primera diligencia q se hade haser es hecharles una, ó dos aiudas, segun fuere el sujeto, y estas hande ser de cosimiento de malbas, un puñado de afrecho labado, con una raíz de bledos machacada, y un pedaso de rapadura, y la sal necesaria sin otra cosa ninguna, y despues de obrada, esto de allí a una ora se le dara un sudor de borrajas, y por la mañana, si la calentura es fuerte, y aviendo las señales dichas

Sangrias =

se le dara una sangría del braso derecho de vena de todo el Cuerpo, y la cantidad q se sacare, sera segun fuere el sujeto, si robusto y mozo quatro, ó sinco onzas; y si devil tres onzas y si niño de uno, ó quatro años dos onzas, y el dia siguiente se le hara sangría del otro brazo en la mesma conformidad = y sobre tarde ala orasion, los quajaran de ventosas

Ventosas =

desde las pantorrillas hasta la punta de las nalgas, y que esten hechadas un quarto de ora = y en quitandolas les daran una friega sobre ellas blandamte y en empesando á apuntar las viruelas los tendran en parte abrigada donde no les de el viento, y q esten abrigados escusando y ará candela en la parte donde estubiere el Enfermo.

Gargarismo

Y porque luego les da mucho dolor de garganta por las viruelas q en ella salen hande haser gargarismo cada rato, y este hade ser cosimiento de sevada cosida hasta q rebiente, y luego se le hande hechar unas ojas de llantén, y en dando un cruar, buscarla en una vasija, y hecharle un poco de sumo de moras, y otro poco de sumo de mansanas verdes y con esto tivio haran gargarismo a menudo.

Ojos

Y si tubieren cargason, y dolor en los ojos se hade atender á ellos con mucho cuidado, porq no crien nube, esto se hace hechandoles cada rato en ellos con una pluma una gota del colirio siguiente = coser unas ojas de llanten, y en una poca de esta agua, hechar un poquito de aceite de guebo, q este se hase batiendo la clara hasta q toda se conbierta en espuma, y luego se pone a escurrir por gran rato y la q escurriere es el aceite, y asi mesmo se tomarán tres ebritas de asafrán de castilla, y se labaran en agua tibia y se moleran muy bien en una cuchara de plata, y de la agua del colirio se hecharán unas gotas para diluirlo, y esto se hechara endicho colirio=

Narises

Y si les salieren Viruelas en las narises, se coseran unas malvas, y en un poca de esta agua se hechara una cucharada de miel rozada, y afalta sera miel, d avejas con media cucharada de almibar en q aya ervido una poquita de rosa y de esta agua sorveran por las narices tivia cada dos, ó tres horas ___

Y si en la lengua salieren viruelas con el gargarismo que queda dicho se templaran___

Agua q hande beber

La agua comun q hande beber hade ser quebrantada y de sebada cosida hasta q rebiente, y en aviendo reventado, se echaran unas hojas de borraja, y unas ramitas de chulco, y sí aí pasas una dosena de ellas quitado los granos, y en dando un ervor con todo esto se apartara___

Comer.

Pueden comer carnero, pollo, y gallina = y sino pudieren comer se les daran unas coladitas de quinua cocida dos veses, y en ella se le hechara una pechuga de ave bien molida = y tambien se les pueden dar coladas de sebada cosida sin cascara = y tal vez se les puede dar un guebo fresco hecho en almíbar y entre comida se les puede dar una tuna, ó un cogollo de lechuga.

Llagas en la boca

Si tubieren llagas en la voca puede tener en ellas un gajo de pína, por q le refresq, y castre, pero q no lo trague, y por q las viruelas despues de aver brotado bien, suelen meterse adentro, aplanarse, de modo q no quedan abultadas,

Quando se entran ô aplanan

y nada de esto es bueno porq suele resultar un trabajo y suele resultar esto de malicia del humor, ó de desabrigo, ó de lebantarse, y de qualquiera causa q sea, es presiso ocurrir al reparo, y esto se hace dandoles á beber el peso de un real de semilla de inojo molida en agua rosada tibia = y otras veses el peso de medio real de piedra besar, molida, y en agua rosada tibia, y en el comedido los restregaran con ortigas fuertes empesando desde los pies por detras y por delante subiendo subiendo para arriba hasta los ombros; y otras veces los cuajaran de ventosas, las primeras desde las pantorillas hasta las puntas de las nalgas, y las segundas en todo el cuerpo hasta q las viruelas buelban a abultarse, q entonces se asegura el enfermo = y sino buelben a abultar con las diligencias dichas, es mala señal.

Alos cinco, ó seis días de brotadas las viruelas estaran ya en salen, y maduras, y entonces se hande rebentar punzandolas con un alfiler de oro, ó de los ordinarios, como no sea de yerro, y despues de rebentadas se hande enjugar blandamte con un pañito tibio, y estandolo se hara una salmuera, no fuerte, y en ella se hechara una cucharada de arina de abas crudas y mandadas, y otra cucharada de arina de lentejas crudas y se pondra a calentar, y con esto untaran en las viruelas con un pañito, y si despues bolvieren á llenar se algunas, se punzaran, y untaran como queda dicho, y despues de hecha esta diligencia, los dejaran hasta q se haran secando, y cayendose las costras, y se advierte q al tiempo de secarse ay peligro de empeorar si hacen algun desman, ó se lebantan al viento.

Cursos

Si les dan cursos estando vrotadas las viruelas, no conbiene dejarlos correr, y ase se les hecharan aiudas, unas veses de almidon tostado en agua azerada, ó de las fraguas de los herreros con un terron de asucar, y despues de apartadas del fuego, se le hecharan dos guebos bien batidos con yema, y clara = y otras veses se haran de sebada tostada machacada, y puesta a coser con media gallina en bastante cantidad de agua q cuesa mucho tiempo hasta q la gallina casi se desbarate, y en estandolo se le hechan un puñado de afrecho labado, un poquito de rosa, y un terron de asucar, y colado esto hecharán dos yemas de guebos bien batidas= y ambas aiudas siempre q se hechen hande ser tibias y así mismo les daran coladas de almidon tostado hechas en agua azerada, y una cucharada de agua rosada, y mientras duraren los cursos, no les daran agua q se á dicho arriba, sino q beban agua azerada, y poca = y si tubieren debilitación, les pondran en el estomago un pedasito de carnero joagado rozeandole unas gotas de vino blanco, y de agua rosada, y se lo quitaran quando este frio, poniendo en su lugar un pañito tivio.

Para la gente delicada en lugar de la salmuera, q se a dicho quando se punzen las viruelas, los podran untar con unguento satrino, y si no lo hubiere, se hara con la salmuera como queda dicho___

Si quedaren mal humorados despues de secar las viruelas podrán purgarlos con onza, y media de maña en agua de vorrajas en q ará dado un ervor la oja de zen q se cogiere con dos dedos___

Adviertase q si se cogiere muy robusto el cuerpo mal humorado en los principios, y se conociere que la calentura esta muy ardiente y con muchos desasosiegos el enfermo, aunq aya tres días, o quatro q broten las viruelas, se le podra dar otras dos sangrias de la vena q se a dicho de todo el cuerpo porq no es de esencia que con dos sangrias se haia de haser la curación, ni tampoco importa que ayan brotado las viruelas para que se deje de sangrar, como no pase del quarto dia, y en rigor de grave necesidad, los podran sangrar, aunq aya sinco días, q brotaron.

Quiera Dios que sea para honrar, y gloria suia, y para que se socorran los pobres que padecen achaq tan penoso, y molesto.

Demas de lo q se refiere en la receta adjunta para curar las viruelas se advierte las cosas siguientes.

Gargarismo

En el gargarismo, afalta de sumo de moras, y mansanas verdes, se puede suplir con el sumo de una lima, ó limon, y q estos gargarismos he hagan desde el principio, y a menudo; y si se hallan puchucululos, es gran medicina para darles deshechos, y con una poca de asucar, q templan famosamte las ansias, q son mortales las q padesen al brotar las viruelas, y esto se da frio, que refrigera, y aun repara el que salgan por dedentro, q es aloq se tira con los gargarismos.

Colirio

El colirio para los ojos, vasta q sea agua fría, y lava, y se le podra añadir el azeite de guebo, que es fácil, y tambien combiene, q desde el principio, si sienten cargazon, y dolor enlos ojos, se heche muy a menudo esta agua tirando a que resuelban y no salgan viruelas en ellos, q suelen segar.

Quando se entran ô aplanan

Aunq falten algunas de las cosas q se disen en la rezeta, lo q importa, si se reconoce q se entran ó aplanan, es tratar de que suden por todos los medios q fuere posible, q con esto buelben a brotar y tenerlos muy abrigados por defuera en todos tiempos y darles cosas frescas por la boca, es regla general.

Estando maduras

No conbiene estando maduras las viruelas cortarlas por mas que los aconsejen, sino q basta con un trapo o paño de lienso tibio, irlas apretando, y las mas rebientan; y donde fuere necesario; lo mas q se permite es un rebentando con un alfiler que no sea de yerro, y luego enjugarlas con el paño tibio, y despues untarlas con un poco de salmuera q no sea muy fuerte, tibia.

Abrigo

El abrigo en todo caso importa mucho desde que empiesan á brotar hasta que estan bien sanos, y q lo q bebieren sea fresco.

Sangrias

Despues de secas las viruelas, quando ya se han descascarando, combienen dos sangrias de la vena de todo el cuerpo, y si han tenido muchas, y quedado mal humorados, que se reconosca q han menester mas, se les podran dar, y luego purgarlos con canafistola y mechoacan y la cantidad conforme a los sujetos”.

## Análisis

Acorde con el desarrollo de la medicina de la época, en la receta es evidente la creencia en la utilidad del agua para sanar la enfermedad y como elemento central de los tratamientos expuestos en función de sus propiedades como desintoxicante y purificadora del cuerpo. En la primera parte del documento, se argumenta que el agua es, en esencia, lo que cura de raíz el mal, más allá de los efectos de los demás componentes referidos. En efecto, en el desarrollo de la receta se logra apreciar la convicción del autor sobre este punto en la siguiente cita: “Pero qué cosa más común que curarse con solo agua, o con poco más que agua, las viruelas”*.* En este orden de ideas, se puede comprender por qué la mayoría de los tratamientos descritos son hechos a base de agua o de diferentes componentes hídricos.

En cuanto a la etiología de la enfermedad, el autor manifiesta abiertamente que se ignora la causa de la viruela y que lo que se hace, en general, es tratar los síntomas visibles en el cuerpo del enfermo. También enuncia que la “cura” verdadera de los males viene de los productos que la naturaleza provee, de tal manera que el quehacer médico se relega a un segundo plano. El papel del saber médico no resulta tan importante como los beneficios de los componentes estrictamente naturales, cuya aplicación se plantea casi *motu proprio*, en calidad de recetas caseras. El mismo autor afirma que, si la enfermedad avanza de manera moderada, “nada tiene que recetar el médico*”*, como se evidencia en la siguiente cita: *“*Es prudencia entonces fiar a la naturaliza todo el negocio, dando a beber según el método*”.*

Las infusiones y mezclas con distintas hierbas, frutas y otras plantas, forman parte esencial del tratamiento de los pacientes con viruela. Sin embargo, todos los tratamientos expuestos solo reducen los síntomas de los pacientes, pero no erradican la causa de la enfermedad. Solo cuando se comprendió la etiología viral específica de estas enfermedades, después del hallazgo e implementación de la escarificación (en el caso de las viruelas), y el advenimiento de la microbiología y la inmunología, se logró abordar una aproximación preventiva.

En varios pasajes se menciona la sangría como tratamiento para los síntomas que provoca el virus en los pacientes. Para la época, la sangría era un procedimiento común que consistía en la extracción de sangre como medio de purificación del cuerpo, de evacuación de impurezas o de “humores” que estaban presentes en exceso, para que se nivelaran hasta llegar a un aparente equilibrio ^7^. En la receta se menciona que la sangría se hace principalmente en los brazos, un día en el derecho y al día siguiente en el izquierdo, y se aclara que la profundidad de la incisión depende de la robustez, la fuerza y la edad del paciente. Queda claro, así, que muchos consideraban la sangre como el torrente por el que circulaban los componentes del cuerpo y, por ello, la sangría se hacía con el fin de controlar la fiebre, eliminar las toxinas, tratar los síntomas de algunas dolencias, incluso si no se conocía su origen, como en el caso del virus de la viruela. Otro efecto de las sangrías sería el de disminuir la inflamación provocada por las ventosas (efecto secundario del tratamiento) y por la infección, lo que en aquella época, naturalmente, no podía clasificarse como enfermedad infecciosa de origen hospitalario, puesto que los enfermos se trataban en sus propias casas.

En el manuscrito se menciona también el uso de los gargarismos a base, una vez más, de agua, los cuales debían hacerse repetidamente para aliviar el malestar de garganta producido por el virus y por la posible aparición de llagas. La mezcla empleada incluía cebada cocida, hojas de llantén (*Plantago major*), zumo de moras y de manzanas verdes o, en su defecto, frutas cítricas como la lima o el limón; cada ingrediente de esta mezcla aportaba alivio al paciente por razones que hoy están claras. La cebada, por ejemplo, tiene efectos antiinflamatorios y desintoxicantes, y el llantén es un cicatrizante y antiinflamatorio útil en la curación de heridas en la piel, cuyo uso está indicado en el tratamiento de enfermedades dermatológicas, respiratorias y gastrointestinales, y también, como analgésico y antioxidante ^8^. Las frutas cítricas, por su parte, disminuyen o, por lo menos, alivian el dolor de garganta.

Se menciona, asimismo, el uso de ventosas sobre el cuerpo del paciente. Según la *Enciclopedia Moderna de Literatura, Ciencias y Arte*, del geógrafo español Francisco de Paula Mellado (1818-1876), la aplicación de ventosas es un método que consiste en provocar un vacío sobre una parte del cuerpo del paciente. En la enciclopedia citada se exponen dos tipos de ventosas: unas secas, que se aplican contra la hinchazón y la rubicundez de la piel, y otras escarificadas, que producen una evacuación de sangre más o menos abundante. Las ventosas sirven para extraer el pus o la sangre acumulada en una herida, para reestablecer el flujo humoral en la superficie de una úlcera o para determinar una irritación que puede comprometer un órgano. En la receta se menciona que es importante que las ventosas se apliquen con protección contra el frío y el viento y que el enfermo se mantenga preferiblemente en una zona caliente ^9^.

Según esta definición, la aplicación de ventosas no era, en sí, un tratamiento directo para tratar la viruela, pero sí contribuía a disminuir las dolencias y los síntomas de los enfermos. En el compendio de recetas se explica el método de aplicación de las ventosas: deben ponerse en el miembro inferior del paciente mientras este permanece acostado en un lugar caliente y protegido del frío durante el tratamiento y después de él.

Como el virus de la viruela afectaba cualquier tejido del cuerpo con el que entrara en contacto, los ojos no estaban exentos de su acción. Las infecciones en los ojos se presentaban en un estadio tardío de la viruela y causaban gran malestar a los enfermos. Aquí de nuevo aparece el agua como método curativo y, en este caso, era la base de los colirios o se empleaba en los paños de agua fría para refrescar la zona de la infección y de la inflamación.

Según la enciclopedia de Mellado, los colirios tienen distintas presentaciones farmacéuticas, en polvos para diluir o en líquido, siendo estos últimos los que más se mencionan en el recetario. En la enciclopedia también se hace una distinción entre los colirios simples y los colirios compuestos, los primeros son aguas destiladas, como las de rosas, de llantén, de hinojo o de hoja de malva, todas ellos eficaces para reducir la inflamación en los ojos; los segundos son preparaciones complejas que, además de tener ingredientes naturales, incluyen elementos más complejos, como el sulfato de cinc. El colirio más conocido y, tal vez, el más eficiente de la época era el de agua de rosas y llantén ^9^. El colirio se empleaba para mitigar el daño que el virus provocaba en la piel de los enfermos y aliviar la sensación de cargazón que producía, así como el daño causado por las vejigas, esperando que se mejoraran y no salieran más, ya que se sabe que podían dejar ciego al paciente.

Es bien conocido que la viruela causaba una serie de llagas o de pústulas en la piel de los infectados, lesiones que solían dejar cicatrices en los sobrevivientes. La receta explica que las llagas debían dejarse madurar para, posteriormente, reventarlas con un alfiler, limpiar con un paño tibio y poner salmuera en el área. Después de reventar las llagas, debían aplicarse hojas de borraja (*Borago officinalis)* y ramas de chulco (*Oxalis curniculata*). La borraja es una planta con propiedades sudoríficas, diuréticas, antiinflamatorias y emolientes, útil en afecciones de las vías respiratorias, y beneficiosa para la piel; además, ayuda a disminuir la inflamación local y la sensación de picazón en la piel, lo que causa cierto alivio en los pacientes. Sin embargo, puede resultar tóxica debido a la presencia de alcaloides pirrolizinídicos, los cuales causan efectos hepatotóxicos y carcinogénicos. El chulco, por su parte, es una planta que disminuye la fiebre y que, en aplicación tópica, se emplea para tratar llagas en los labios, encías y lengua y, también, como ingrediente para hacer gargarismos ^10^^,^^11^.

Mencionado también en las recetas, el llantén tiene diversos efectos terapéuticos que se relacionan a continuación con base en dos referencias bibliográficas contemporáneas ^12^^,^^13^: como desinflamante y antiinflamatorio, sus hojas deben hervirse y estando tibias, colocarse como emplastos en la parte afectada; como remedio pectoral, por su contenido de ácido silícico y mucílagos; como cicatrizante, ya que estimula la regeneración y proliferación celular en el sitio de la lesión; contra las úlceras y las quemaduras en compresas; para aliviar las anginas en gargarismos y en colirio; para la conjuntivitis y la inflamación en los párpados; como astringente y para tratar quemaduras, enfermedades de la piel, picaduras de insectos, irritación en los ojos e inflamaciones en boca y garganta; como alivio de la irritación en las vías respiratorias superiores, y como antibiótico y antibacteriano.

Algunos de estos usos se mencionan en repetidas ocasiones en las recetas: en el tratamiento de los efectos de la viruela en la piel de los infectados, puesto que es desinflamante; como cicatrizante y para aliviar las anginas con gargarismos, y para la conjuntivitis y la inflamación de los párpados si se usa como ingrediente en los colirios. Sus hojas son astringentes y sirven para tratar quemaduras y áreas inflamadas y en las recetas se explica su uso en paños de agua tibia después de reventar las pústulas de la viruela.

Entre los documentos españoles y neogranadinos de la época con descripciones de la enfermedad, cabe mencionar el informe de 1782 de José Celestino Mutis titulado *Instrucciones sobre las precauciones que deben observarse en la práctica de la inoculación de la viruelas, formada de orden del superior Gobierno*, así como lo reportado en el *Tratado de medicina práctica* (1851) de Peter Frank, *et al.*^14^, en el cual se define la viruela como un exantema primitivo y contagioso, cuya erupción es precedida de fiebres, náuseas, vómitos y convulsiones en los niños, y sudor en los adultos. Al tercer o cuarto día, aparecen en la piel unas manchas rojizas con un punto duro en el centro, las cuales evolucionan a pústulas y vejigas que se llenan de una materia incolora. Si se procede a la desecación, lo que correspondería a reventarlas, dejan una cicatriz deprimida e hipercrómica en el lugar. La viruela puede ser discreta, con pústulas aisladas, o confluente, con pústulas en racimos. En este tratado, así como en el recetario del Archivo Histórico que se comenta, se evidencia la creencia de que el humor y el estado de ánimo del paciente serían la razón de ser de los síntomas de la viruela, pero en el tratado no se menciona que las llagas de la viruela aparezcan en el interior del organismo, por lo que este tipo de condiciones puede pasar desapercibido y suele causar un exceso de saliva.

El curso clínico de la viruela se divide en cuatro periodos: el primero es el de la invasión, que es el tiempo más favorable para el uso de remedios; el segundo es el de la erupción; el tercero se caracteriza por la supuración y, durante el cuarto, ocurre la desecación. Esta misma evolución temporal se aplicaría a los síntomas del sarampión, cuyos efectos clínicos y causas específicas no están diferenciados en las fuentes contemporáneas a la receta que se comenta aquí. Para una contextualización del manejo terapéutico y el entorno social de estas virosis en el Nuevo Reino de Granada, y para ahondar en el sentido de la sangría como recurso principal, se pueden consultar otras fuentes historiográficas en las que se ha tratado el tema con autoridad ^15^^-^^18^.

## Conclusión

En función de la progresiva obtención y suma de conocimientos, y con la llegada de nuevas tecnologías y prácticas unificadas a todos los rincones del planeta, el tratamiento de las enfermedades ha avanzado con relativa rapidez y los del pasado se han visto relegados a curiosidades históricas de carácter anecdótico. Sin embargo, debe considerarse que aquellos más o menos antiguos, como los expuestos a lo largo del presente artículo, permiten comprender mejor la terapéutica precientífica y, en algunos casos, promoverlos como recursos terapéuticos para aliviar la sintomatología de algunas enfermedades. El legado en las fuentes primarias conservadas en los archivos históricos puede iluminar, así, a los nuevos practicantes de la medicina y a los científicos contemporáneos, y darles la oportunidad de viajar al pasado para intentar comprender las costumbres de quienes nos precedieron.

## Fuentes primarias:

Archivo Histórico Cipriano Rodríguez Santa María, Fondo Manuel María Mosquera, Universidad de La Sabana: https://intellectum.unisabana.edu.co/ handle/10818/18140
